# The Use of Primary Care Electronic Health Records for Research: Lipid Medications and Mortality in Elderly Patients

**DOI:** 10.3390/pharmacy7030134

**Published:** 2019-09-18

**Authors:** Adam J. Hodgkins, Judy Mullan, Darren J. Mayne, Andrew Bonney

**Affiliations:** 1 Graduate Medicine, Faculty of Science, Medicine and Health, University of Wollongong, Wollongong 2522, Australia; 2 Illawarra Health and Medical Research Institute, University of Wollongong, Wollongong 2522, Australia; 3 Centre for Health Research Illawarra Shoalhaven Population, University of Wollongong, Wollongong 2522, Australia; 4 Public Health, Illawarra Shoalhaven Local Health District, Warrawong, NSW 2502, Australia; 5 Sydney School of Public Health, University of Sydney, Sydney 2006, Australia

**Keywords:** hypolipidemic agents, family practice, electronic health records, survival analysis, aged, aged 80 and over

## Abstract

General practice electronic health record (EHR) data have significant potential for clinical research. This study demonstrates the feasibility of utilising longitudinal EHR data analysis to address clinically relevant outcomes and uses the relationship between lipid medication prescription and all-cause mortality in the elderly as an exemplar for the validity of this methodology. EHR data were analysed to describe the association of lipid medication use, non-use or cessation with all-cause mortality in patients aged ≥75 years. Survival analysis with Cox regression was used to calculate hazard ratios, which were adjusted for confounders. There was no significant difference in all-cause mortality among patients according to their use, non-use, or cessation of lipid medications. The outcomes of this study correlate well with the results of other research works. This single-practice study demonstrates the feasibility and potential of analysing EHR data to address important clinical issues such as the relationship between all-cause mortality and lipid medication prescription in the elderly.

## 1. Introduction

The substantial volume of data which exist in general practice electronic health records (EHRs) presents opportunities for clinically relevant primary care research [1]. These research opportunities are further enhanced because data are collected about patients, their health conditions and their treatments within the environment in which they occur, thereby providing an important contextual factor to the research [2]. The use of data recorded during routine care in primary care settings, rather than collected under experimental conditions, is appropriate for the evaluation of comparative safety or effectiveness of management [3]. The use of data collected from EHRs in pragmatic trials and comparative effectiveness studies is an emerging research method [4]. Whilst randomised controlled trials are important for informing clinicians of the ability of an intervention to make a difference in ideal circumstances (efficacy), pragmatic trials are needed to measure the degree of benefit in a real-world setting (effectiveness).

Analysis of data collected routinely in the course of providing health care has a valuable place in general practice research [5]. Data recorded contemporaneously can overcome recall bias [6]. By using EHR data, the time and cost of additional data collection can be reduced [4,7]. While some data in EHRs are of variable quality, other data are of high quality and have been benchmarked against accepted general practice standards [8].

General practice data have the potential to address important health outcomes which are considered by general practitioners (GPs). For example, the examination of the outcomes related to prescribing patterns can inform future practice [9]. For instance, lipid-lowering medications such as statins, are amongst the most frequently prescribed medications in Australia [10]. The risks associated with statins increase with advancing age and with polypharmacy, which is common in older patients [11]. It is important, therefore, to gain a better understanding about outcomes for elderly patients in relation to the prescribing of these medications by general practitioners.

Currently in Australia, the Heart Foundation recommends the use of absolute cardiovascular risk to guide decisions regarding the use of lipid-lowering therapy as a primary prevention [12]. The latest guidelines suggest that all patients over 75 years of age can be assessed using the same risk algorithms designed for those aged between 45 and 75. Notably, however, this recommendation is supported by an expert consensus statement rather than literature describing well-designed trials [13]. Much of the available literature suggests that there may be reductions in cardiovascular events when statins are prescribed to the elderly as secondary prevention. However, the evidence is lacking for primary prevention in this age group, and there is a paucity of evidence to suggest that statins influence mortality [14,15,16,17,18].

Thus, there is a need for further research into the outcomes for elderly patients prescribed lipid-lowering medications [18], and the research question is well-suited for primary care research using a retrospective cohort design. The data required for such a study are accurately recorded in general practice EHRs, and the potential to study a large number of subjects over a relatively long period of time is easily realised [19]. The objective of this study was to demonstrate the feasibility of using longitudinal EHR data for research and quality improvement, using the relationship between lipid- lowering medications and all-cause mortality among elderly patients as an exemplar.

## 2. Materials and Methods

EHR data were drawn from a general practice in regional New South Wales, Australia. This practice had, on average, nine GPs working during the study period. The EHR in use was Best Practice Software™ [20], which uses a structured query language (SQL) database to organise relevant patient data. An SQL query was developed (by the primary author, AH) to obtain de-identified data relating to eligible patients.

Data were considered eligible if the patient attended the practice between 1 January 2007 and 31 December 2015 and was ‘active’ (defined as having a minimum of three visits in the two years prior to study entry) [21]. Entry to the study was the latter of: 1 January 2007; the patient’s 75th birthday; or one year after their first practice visit. The twelve-month lead-in period was determined, as patient data including usual medications and past medical history are often not complete at the first visit but added to the EHR at subsequent consultations.

Data were censored on 31 December 2015 for patients seen after this date, or on the date of their last visit if not seen subsequent to 2015. For all data which were not censored, the study outcome was the date of death recorded in the EHR.

The data collected included GP visit dates, dates of birth and death, prescription data, smoking and marital status, and history of vascular disease or diabetes. Patients’ names and addresses were not retrieved to ensure the data remained de-identified.

Patients were classified according to their history of lipid-lowering medication prescriptions. ‘Non-Users’ had no record of prescription for lipid-lowering medications, including prior to study entry. ‘Users’ had been prescribed lipid-lowering medications, and their last recorded prescription was less than 12 months prior to death or censoring. ‘Stoppers’ had been prescribed lipid-lowering medications but had not received a prescription for at least a year prior to death or censoring.

The all-cause mortality risks of these three groups of patients were compared after adjustment for possible confounders using Cox regression, with non-users as the reference category. Possible confounders which were adjusted for included age at study entry, number of prescriptions for any medication per year (previously demonstrated as a proxy for multimorbidity) [22,23], smoking status at the end of the study, marital status and the presence of ischaemic heart disease, cerebrovascular disease, peripheral vascular disease or diabetes. We also undertook subgroup analyses stratified by treatment for either primary prevention (i.e., no history of vascular disease) or secondary prevention (patients with a history of one or more cardiovascular, cerebrovascular or peripheral vascular disease, or diabetes). Data analysis was performed with SPSS [24] using two-tailed significance tests and a type 1 error rate of 0.05.

This research was approved by the Joint University of Wollongong and Illawarra Shoalhaven Local Health District Human Research Ethics Committee (ref-2014/434).

## 3. Results

Data from a total of 1911 patients in the practice EHR database were eligible for study entry. Of these, 324 (16.9%) were missing the smoking status, and 318 (16.6%) were missing the marital status. In total, 431 (22.6%) were excluded due to missing data on one or more of the outcome variables, study variables, or adjustment variables, giving an analytic data set of 1480 (77.4%) patients. The excluded data had similar demographics and outcomes as the analytic data. The characteristics of the participants are shown in Table 1.

The median duration of follow-up was 4.50 years. Overall, 6840 patient-years of data were examined, and the outcome of death from any cause was measured in 265 patients (17.9%), with the remainder being censored. The age at study entry ranged from 75 to 102 years, with the median of 78.02 years of age. The number of prescriptions for any medication ranged from 0 to 135 per year, with a mean of 19.4 prescriptions per year. Statins comprised 95.0% of all lipid-lowering medications prescriptions in this sample, with fibrates and ezetimibe making up the remainder. There were no significant differences in outcomes for those prescribed statins compared with those prescribed other lipid-lowering therapies.

Cox regression was used to calculate the hazard ratios (HR) of the variables, both unadjusted and adjusted for the other variables examined. These HRs are shown in Table 2.

Increasing age, male gender, current smoking and increasing numbers of prescriptions per year were all associated with a statistically significant increased hazard for all-cause mortality. There was no increased risk of mortality for patients who had their lipid medication ceased nor for those patients who had never been prescribed lipid medication. This was true also for the subset of patients with a history of vascular disease or diabetes, whose lipid medications could be classed as secondary prevention. A trend for improved survival among primary prevention patients who stopped taking lipid medication relative to continuous users did not achieve statistical significance.

**Figure 1:**
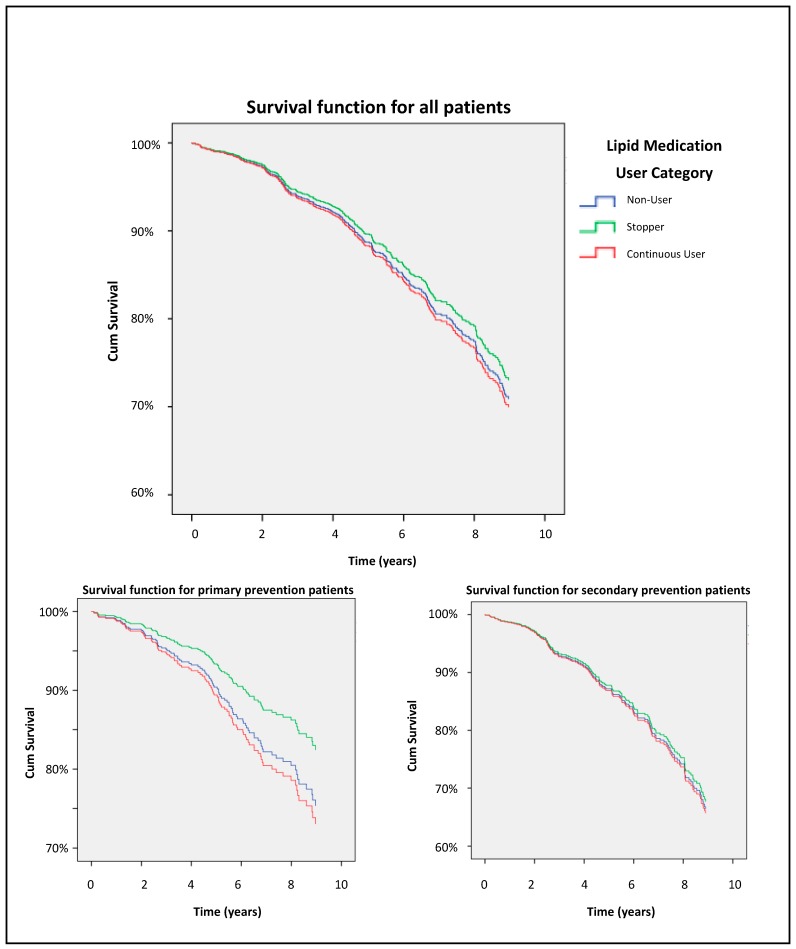
Survival function curves (adjusted for confounders).

Adjusted Kaplan–Meier curves shown in Figure 1 demonstrate the non-significant differences in survival for the different groups based on lipid medication use.

## 4. Discussion

This study design was able to address important primary care clinical outcomes using data recorded contemporaneously during the provision of routine care. The examination of EHR data using survival analysis to achieve a longitudinal design offers a novel approach and carries significant advantage over cross-sectional studies when examining the outcomes of differing treatment decisions [25].

Our research was unable to demonstrate any increased risk of death associated with cessation of lipid medications in elderly patients. We were unable to find other research specifically examining mortality in older patients in relation to lipid therapy cessation. The separation of the Kaplan–Meier curves between stoppers and other lipid medication groups is interesting and supports powering future EHR studies for further analysis.

Although we found no survival advantage in patients treated for secondary prevention, other studies examining mortality and lipid therapy in older patients found a small survival advantage in elderly patients with cardiovascular disease [26] or with diabetes [27], suggesting further subgroup analysis may be beneficial.

Our research is consistent with other research using prospective data which also shows no survival advantage when lipid medications are used for primary prevention in the elderly [17,27,28].

Known risk factors such as increasing age, male gender, prescription counts and current smoking were associated with increased hazard for all-cause mortality. Despite incomplete smoking data in the eligible patients, our research demonstrated an adjusted hazard ratio of 2.9 which correlates well with the results of another study which demonstrated relative mortality rates in older smokers of 1.2 to 3.4 [29]. This suggests that the study design can identify such risk when present.

A limitation of this study is that the use of data from a single practice limits the generalisability of the results. A wider data collection with greater numbers and broader geographical and socioeconomic spread will enable a more extensive application of the results. The data were originally recorded to facilitate clinical care rather than for research purposes. No information is known about the reasons for ceasing lipid medication or even not prescribing it. If patients with less severe disease are more likely to have medication ceased, it may bias the results toward greater survival in this group. Conversely, if lipid medications are ceased because the clinician perceives a shortened lifespan due to comorbidities, the bias would be reversed. Missing data regarding smoking and marital status have the potential to bias the results. Further examination of the patients excluded due to missing data will be necessary on a larger sample size to more accurately determine if a bias exists. The duration of confounders such as a diagnosis of diabetes or vascular disease was not measured, nor was the duration or type of lipid medications quantified in our study. Further analysis of these factors in future studies would be interesting and suited to this study design.

Analysis of EHRs has been employed to examine the relationship of statins with mortality in the elderly in a large study in Spain [27]. Further research using a study design similar to that of our study but with a larger volume and spread of data will be helpful to make comparisons with the existing international research. Additionally, further research using this methodology has the potential to address a common fear of deprescribing lipid medications to elderly patients [30]. This study design has demonstrated the validity of longitudinal analysis of EHRs at a practice level and offers a low-cost method of examining real-world primary care data to address important clinical questions.

## Figures and Tables

**Table 1:** Characteristics of the study population.

	Continuous Variables (Mean ± Standard Deviation)				
	Excluded Cases	Complete Cases	Non-User	Stopper	Continuous User
Age at commencement of study (years)	81.36 (±6.12)	79.15 (±4.83)	80.54 (±5.48)	78.89 (±4.07)	77.65 (±4.07)
Duration of follow-up (years)	2.90 (±2.66)	4.62 (±3.06)	4.45 (±3.03)	5.63 (±2.83)	4.36 (±3.09)
Prescriptions per year	19.87 (±24.93)	19.41 (±16.47)	18.62 (±17.71)	18.45 (±16.92)	20.78 (±14.59)
	Categorical Variables (number, percentage)				
	Excluded Cases	Complete Cases	Non-User	Stopper	Continuous User
**Outcome**					
Death	66 (15.3%)	265 (17.9%)	131 (19.8%)	51 (20.0%)	83 (14.7%)
Censored	365 (84.7%)	1215 (82.1%)	529 (80.2%)	204 (80.0%)	482 (85.3%)
**Gender**					
Male	173 (40.2%)	641 (43.3%)	292 (44.2%)	91 (35.7%)	258 (45.7%)
Female	257 (59.8%)	839 (56.7%)	368 (55.8%)	164 (64.3%)	307 (54.3%)
**Smoking status**					
Non-smoker	40 (37.0%)	645 (43.6%)	279 (42.3%)	119 (46.7%)	247 (43.7%)
Former smoker	60 (56.5%)	780 (52.7%)	355 (53.8%)	124 (48.9%)	301 (53.3%)
Current smoker	7 (6.5%)	55 (3.7%)	26 (3.9%)	12 (4.7%)	17 (3.0%)
**Marital status**					
Married or de facto	41 (36.9%)	763 (51.6%)	314 (47.6%)	124 (48.6%)	325 (57.5%)
Single or separated	28 (25.2%)	159 (10.7%)	79 (12.0%)	20 (7.8%)	60 (10.6%)
Widowed	42 (37.8%)	558 (37.7%)	267 (40.5%)	111 (43.5%)	180 (31.9%)
**Cardiovascular disease**					
No	348 (80.7%)	1062 (71.8%)	573 (86.8%)	154 (60.4%)	335 (59.3%)
Yes	83 (19.3%)	418 (28.2%)	87 (13.2%)	101 (39.6%)	230 (40.7%)
**Cerebrovascular disease**					
No	380 (88.2%)	1226 (82.8%)	570 (86.4%)	195 (76.5%)	461 (81.6%)
Yes	51 (11.8%)	254 (17.2%)	90 (13.6%)	60 (23.5%)	104 (18.4%)
**Peripheral vascular disease**					
No	421 (97.7%)	1400 (94.6%)	640 (95.5%)	235 (92.2%)	525 (92.9%)
Yes	10 (2.3%)	80 (5.4%)	30 (4.5%)	20 (7.8%)	40 (7.1%)
**Diabetes mellitus**					
No	361 (83.8%)	1163 (78.6%)	584 (88.5%)	173 (67.8%)	406 (71.9%)
Yes	70 (16.2%)	317 (21.4%)	76 (11.5%)	82 (32.2%)	159 (28.1%)
**Lipid medication use**					
Never	286 (66.4%)	660 (44.6%)	660 (100%)	-	-
Ceased	54 (12.5%)	255 (17.2%)	-	255 (100%)	-
Current	91 (21.1%)	565 (38.2%)	-	-	565 (100%)

**Table 2:** Unadjusted and adjusted hazard ratios for all-cause mortality (HR, hazard ratio; REF, reference; CI, confidence interval).

	All Patients		Primary Prevention		Secondary Prevention	
	Unadjusted HR (95% CI)	Adjusted HR (95% CI)	Unadjusted HR (95% CI)	Adjusted HR (95% CI)	Unadjusted HR (95% CI)	Adjusted HR (95% CI)
Age (per year)	1.12 (1.09–1.14)	1.14 (1.12–1.17)	1.12 (1.08–1.15)	1.13 (1.08–1.17)	1.13 (1.09–1.16)	1.15 (1.11–1.19)
Gender						
Female	REF	REF	REF	REF	REF	REF
Male	1.30 (1.05–1.61)	1.61 (1.22–2.11)	1.13 (0.76–1.70)	1.46 (0.92–2.32)	1.38 (1.01–1.87)	1.63 (1.20–2.40)
Smoking status						
Non-smoker	REF	REF	REF	REF	REF	REF
Former smoker	0.92 (0.72–1.18)	0.74 (0.57–0.96)	0.84 (0.56–1.27)	0.63 (0.40–1.00)	0.93 (0.68–1.27)	0.79 (0.56–1.10)
Current smoker	2.23 (1.34–3.71)	2.91 (1.71–4.95)	3.50 (1.76–6.95)	4.58 (2.16–9.75)	1.41 (0.65–3.07)	2.04 (0.91–4.59)
Marital status						
Married or de facto	REF	REF	REF	REF	REF	REF
Single or separated	0.64 (0.39–1.05)	0.49 (0.29–0.81)	0.58 (0.25–1.36)	0.40 (0.16–0.99)	0.69 (0.37–1.26)	0.52 (0.28–0.97)
Widowed	1.08 (0.84–1.38)	0.77 (0.57–1.02)	1.15 (0.77–1.72)	0.65 (0.40–1.06)	1.06 (0.77–1.46)	0.85 (0.59–1.22)
Prescriptions per year	1.02 (1.02–1.03)	1.02 (1.02–1.03)	1.02 (1.01–1.03)	1.02 (1.01–1.03)	1.02 (1.01–1.03)	1.02 (1.01–1.03)
Cardiovascular disease						
No	REF	REF			REF	REF
Yes	1.33 (1.04–1.70)	1.20 (0.91–1.58)	–	–	1.25 (0.91–1.72)	1.09 (0.76–1.57)
Cerebrovascular disease						
No	REF	REF			REF	REF
Yes	1.24 (0.94–1.65)	1.16 (0.87–1.55)	–	–	1.13 (0.82–1.55)	1.09 (0.76–1.55)
Peripheral vascular disease						
No	REF	REF			REF	REF
Yes	1.25 (0.82–1.90)	1.15 (0.75–1.78)	–	–	0.56 (0.74–1.75)	1.18 (0.75–1.84)
Diabetes mellitus						
No	REF	REF			REF	REF
Yes	0.92 (0.68–1.24)	0.92 (0.67–1.26)	–	–	0.78 (0.56–1.08)	0.89 (0.62–1.28)
Lipid medication use						
Current	REF	REF	REF	REF	REF	REF
Never	1.32 (1.01–1.74)	0.97 (0.70–1.33)	1.57 (0.94–2.64)	0.91 (0.57–1.57)	1.44 (1.01–2.05)	0.97 (0.66–1.45)
Ceased	1.01 (0.71–1.42)	0.87 (0.61–1.25)	0.92 (0.43–1.94)	0.59 (0.26–1.30)	1.05 (0.71–1.56)	0.92 (0.62–1.38)
